# Recent Advances in Genetic Tools for *Acinetobacter baumannii*

**DOI:** 10.3389/fgene.2020.601380

**Published:** 2020-12-22

**Authors:** Ellen M. E. Sykes, Soumya Deo, Ayush Kumar

**Affiliations:** Department of Microbiology, University of Manitoba, Winnipeg, MB, Canada

**Keywords:** multi-drug resistance, gene deletions, genetic complementation, selection marker, counter-selection marker, non-clinical antibiotics

## Abstract

*Acinetobacter baumannii* is classified as a top priority pathogen by the World Health Organization (WHO) because of its widespread resistance to all classes of antibiotics. This makes the need for understanding the mechanisms of resistance and virulence critical. Therefore, tools that allow genetic manipulations are vital to unravel the mechanisms of multidrug resistance (MDR) and virulence in *A. baumannii*. A host of current strategies are available for genetic manipulations of *A. baumannii* laboratory-strains, including ATCC^®^ 17978^TM^ and ATCC^®^ 19606^T^, but depending on susceptibility profiles, these strategies may not be sufficient when targeting strains newly obtained from clinic, primarily due to the latter’s high resistance to antibiotics that are commonly used for selection during genetic manipulations. This review highlights the most recent methods for genetic manipulation of *A. baumannii* including CRISPR based approaches, transposon mutagenesis, homologous recombination strategies, reporter systems and complementation techniques with the spotlight on those that can be applied to MDR clinical isolates.

## Introduction

*Acinetobacter baumannii* is a Gram-negative opportunistic pathogen causing disease in critically ill patients. It exhibits widespread resistance to all classes of antibiotics leading the World Health Organization (WHO) to classify it as a top priority for research (Tacconelli et al., 2018). This necessitates understanding the mechanisms of multi-drug resistance (MDR) and virulence. Tools that allow genetic manipulations are the key to unravel such mechanisms in *A. baumannii*. A host of current strategies are beneficial in manipulation of *A. baumannii* laboratory-strains, including ATCC^®^ 17978^TM^ and ATCC^®^ 19606^T^, but may be insufficient when targeting strains obtained from hospitals and patients more recently, primarily due to their high resistance to antibiotics and the genomic diversity of *A. baumannii* (Diancourt et al., 2010; Zarrilli et al., 2013). Recently, genetic tools including the use of non-clinical antibiotic markers as well as those utilizing non-antibiotic markers have been generated for clinical strains of *A. baumannii* (Trebosc et al., 2016, 2019; Ducas-Mowchun et al., 2019; Lucidi et al., 2019). Many of these tools provide desirable traits in gene manipulation, including unmarked and scarless gene deletions, and complementation that mimic natural systems with no pleiotropic effects. This review compiles challenges with designing genetic tools for antibiotic resistant clinical isolates of *A. baumannii* as well as new developments in the field including high-throughput screening and genome editing such as CRISPR. More routine techniques such as homologous recombination and complementation are covered in excellent depth by Indranil Biswas and we direct the readers to this review (Biswas, 2015). Our review highlights techniques that have evolved since then with a focus on selection and counter-selection markers developed specifically for use in novel MDR and XDR clinical strains of *A. baumannii*, an ever-emerging concern in *Acinetobacter* genetics.

## High-Throughput Genetic Screening

### Transposon Mutagenesis

**FIGURE 1:**
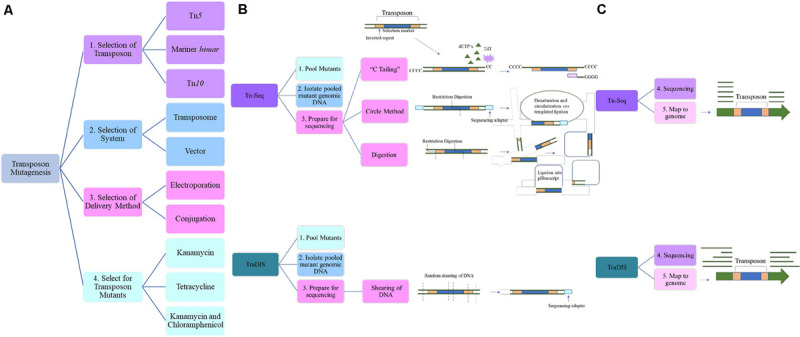
An overview of the steps and considerations when undertaking transposon mutagenesis. **(A)** Considerations for transposon mutagenesis and Tn-seq. During the selection of the transposon, the type should be taken into consideration and in *A. baumannii* Tn*5*, Tn*10*, and mariner *himar* have been successful. The determination of the transposon system is the next step and includes both plasmid or transposome based methods and this will play a role in the method of delivery to the strain of interest. Following this, the selection marker must be considered, and susceptibility profile of the strain taken into account. **(B)** After the mutant library has been selected for and the pooled mutant genomic DNA extracted, the library must be prepared for sequencing. There are three methods of sequencing preparation for Tn-seq. “C-tailing” involves the addition of dCTPs and the terminal deoxynucleotidyl transferase (TdT) and a poly-C tract is added to the 3′ ends of the fragmented DNA. The circle method involves the restriction digestion of pooled DNA using an enzyme that digests within the transposon as well as the addition of a sequencing adapter. This is then circularized *via* templated ligation. Lastly, random restriction digestion of the DNA is followed by ligation of the varying length fragments into a vector such as pBluescript. **(C)** Next-generation sequencing (NGS) techniques are then used. Mapping of the reads to the genome, show an absence of reads where the transposon integrated into the gene and thus allows for determination of insertion.

Transposon mutagenesis allows for global genetic information of an organism to be collected. Mutagenesis studies remain the mainstay in determining functional correlation with genotype (Russo et al., 2016). Three transposons used in *A. baumannii* are Tn*5*, Tn*10*, and *himar1* mariner (Figure 1A). Tn*5* and Tn*10* integrate at non-specific target sites (Bender and Klecknert, 1992; Goryshin et al., 1998) typically minimizing insertion bias compared to mariner transposons whose insertion targets AT dinucleotides (Chiang and Rubin, 2002). Ideally saturation of insertion is achieved, meaning that for every possible insertion site, a transposon mutant exists. As the GC content of *A. baumannii* is around 40%, it presents a bias for mariner insertion lending advantages to the use of Tn*5* or Tn*10* (Chao et al., 2016). Due to the random integration of Tn*5* there is a higher degree of saturation in *A. baumannii* (Gallagher, 2019). However, maximum library density is easily calculated for *himar1* mariner due to its known target site, whereas this is more of a challenge with Tn*5* as there are integration hotspots (Green et al., 2012), resulting in insertion bias with specific sites being overrepresented. It is critical to validate at least two insertion sites per gene to ensure no phenotype is overlooked (Gallagher et al., 2015). Most mutagenesis studies consist of the same basic strategies. An insertion library is generated via delivery of transposon and its cognate transposase of choice resulting in a single cell with one insertion within a population. The mutants are pooled and those mutants with disrupted genes altering fitness under appropriate conditions are selected for or against (Figure 1).

Most commonly, Tn*5* saturated libraries have been generated with the EZ-Tn*5*^TM^ kit (Lucigen Biotechnologies, Middleton, WI, United States) in a wide range of strains, namely, AB5075 (Gallagher et al., 2015; Tipton et al., 2017; Pérez-Varela et al., 2020), BAL062 (Hassan et al., 2016), AB0057 (Crépin et al., 2018), and ATCC^®^ 17978^TM^ (Subashchandrabose et al., 2015). The transposome, composed of the transposon DNA sequence and transposase bound to the 19 base pair mosaic end recognition sequence, are commercially pre-assembled requiring less upstream genetic engineering steps which is beneficial. The selection marker within the transposon is customizable. Electroporation into competent cells allows transposome entry (Gallagher, 2019) however, this step cannot be easily performed in a high through-put manner leading to a preference for conjugation-based protocols (Kazi et al., 2020). One vector-based Tn*5* systems also exist; pRL27 system in ATCC^®^ 17978^TM^ (Kim et al., 2019) which can either be delivered to *A. baumannii* through electroporation or conjugation. Kim et al. mutagenized ATCC^®^ 17978^TM^ and screened for loss of biofilm formation leading to the establishment of BfmRS two-component system’s role in outer membrane vesicle (OMV) production (Kim et al., 2019).

Mariner (Di Venanzio et al., 2019; Kazi et al., 2020; Lopez et al., 2020; Sun et al., 2020) and Tn*10* (Giles et al., 2015; Geisinger et al., 2019, 2020; Roy et al., 2020) transposons also prove useful, in *A. baumannii*. The suicide vector pJNW684 containing the *himar1* mariner transposase, transposon and RP4/oriT/oriR6K origin of replication requires the *λpir* gene product for replication (Wang et al., 2014). Delivery to *A. baumannii* occurs via bi-parental mating with an *Escherichia coli* diamenopimelic acid (DAP) auxotroph which allows for selection of recipient cells (Wang et al., 2014; Kazi et al., 2020). This vector also contains the *Acinetobacter* specific *rpoD* promoter to control transposase expression. A different mariner-based system, pSAM::OmpAp+Tn903, has been applied to ATCC^®^ 17978^TM^ (Di Venanzio et al., 2019) and the Canadian clinical isolate, AbCAN2 (Lopez et al., 2020). An AbCAN2 transposon mutant library provided the basis for determining the essentiality of the Vgr spike protein in the type VI secretion system (T6SS) and its role in interbacterial competition (Lopez et al., 2020). A similar library in ATCC^®^ 17978^TM^ laid the foundation for the discovery that suppression of the T6SS allows for dissemination of MDR plasmids within *A. baumannii* but also within the *Acinetobacter* species itself (Di Venanzio et al., 2019). Tn*10* delivery *via* either electroporation or conjugation, pBSL180 (Roy et al., 2020) pDL1073 (Geisinger et al., 2019, 2020), and pLof (Giles et al., 2015) have successfully generated libraries for *A. baumannii.* The use of pBSL180 (Alexeyev and Shokolenko, 1995) has been marked in other Gram-negatives such as *E. coli* (Scaletsky et al., 2005), *Shenwanella oneidensis* and *Desulfovibrio desulfuricans* (Groh et al., 2005). In ATCC^®^ 17978^TM^, the role of carboxy-terminal processing protease in motility and membrane integrity was characterized (Roy et al., 2020). A drawback of using plasmid-based systems is the risk of integration of the backbone into the mutant chromosome. This can be mitigated by the use of the conditional origin of replication ensuring that the transposase is not maintained in *A. baumannii* (Kazi et al., 2020) which has been engineered into these common systems.

After transposition, insertion mutants are isolated on selective media (Figure 1A). An antibiotic marker is engineered within the transposon that allows for the selection of a successful integration event. Currently tetracycline (Pérez-Varela et al., 2020), kanamycin (Gallagher et al., 2015; Hassan et al., 2016; Geisinger et al., 2019; Kazi et al., 2020; Sun et al., 2020), and a combination of kanamycin and chloramphenicol (Kim et al., 2019; Lopez et al., 2020) are used during library generation in *A. baumannii*. These selection markers have been used in a variety of clinical isolates including AB5075, AbCAN2, BAL062, and AB0057 (Supplementary Table 1). As these isolates remain susceptible to tetracycline, kanamycin and chloramphenicol, the requirement for non-antibiotic or non-clinical drug selection is negligible. However, selection conditions become increasingly critical factors to consider as the diversity and resistance profile of *A. baumannii* is hyper-plastic. Following this, a critical step in library establishment is the storage of the mutants. To ensure that unnecessary selection pressures are avoided, freezing of the pooled mutants must occur very shortly after selection (Kazi et al., 2020). However, as research progresses, storing and exposing strains of interest to new conditions/stress allows investigation of novel effects as demonstrated for an XDR clinical lung isolate HUMC1 (Luna et al., 2020). The authors repurposed a previously generated mutant library in AB5075 in order to validate results observed in HUMC1 (Gallagher et al., 2015). A screen for susceptibility of HUMC1 to a small molecule library in the absence of rich media was undertaken. A notable hit, resulting in an increase in susceptibility to rifabutin, led to the investigation of the molecular mechanism in HUMC1 and also in AB5075. Transposon mutant libraries demonstrate their versatility in the foundation of studies and as demonstrated by Luna et al. (2020) can be repurposed years in the future to investigate and validate novel findings.

### Transposon-Sequencing (Tn-Seq)

Transposon mutagenesis is coupled with downstream steps for further characterization of the mutant library, making it the backbone of many genetic studies (Figure 1B). With the advent of Next-Generation-Sequencing (NGS), it is less cumbersome to sequence the region of integration, and the whole genome, identifying the disrupted genes. Generally, genomic DNA of the pooled mutants is extracted and NGS is used to sequence the transposon integration site and these reads are mapped to the genome. This approach means that mutants with fitness defects are under-represented due to lack of survival. However, this challenge can be overcome by generating conditional libraries, one exposed to particular *in vitro* or *in vivo* conditions, for example and the other generated without any selective pressure. A recent study characterized phenotypic responses to ciprofloxacin in ATCC^®^ 17978^TM^ by comparing those pooled transposon mutants exposed and those untreated (Geisinger et al., 2019). To elucidate the interplay in ciprofloxacin resistance mechanisms, the most common fluoroquinolone resistance alleles, those in *gyrA* and *parC*, were generated. These became the parent strains for the transposon mutagenesis study. A target-dependent prophage and SOS response under fluoroquinolone stress conditions was observed which characterizes the hierarchy of induced resistance to ciprofloxacin.

Following mutagenesis, genomic DNA of the pooled mutants is extracted and sheared. A method known as “C-tailing” facilitates the sequencing procedure (Klein et al., 2012). It involves the addition of poly(C) tails to the 3′ end of the fragmented DNA via the use of dCTPs and terminal deoxynucleotidyl transferase (TdT). A round of PCR enriches the insertion sequences *via* a primer with a 5′ poly(G) tract that binds the poly(C) region previously added and a region that binds the transposon (Gallagher, 2019). Alternatively the use of a protocol originally developed in *Pseudomonas aeruginosa*, the circle method allows for selection of transposon insertion sites (Gallagher et al., 2011). In this technique, after size selection, a sequencing adapter is ligated onto the end of the transposon and circularized. Any remaining uncircularized and denatured fragments, which is the majority, are degraded. A final PCR step amplifies the transposon junction, initial sequencing primer and introduces the second required sequencing primer. Validating this approach, Gallagher et al. (2015) developed a three-allele library using both the circle and terminal deoxynuclease transferase methods of sequencing preparation, generating similar libraries and demonstrating that any amplification-based bias in the circle method is negligible. Alternate sequence preparation includes digestion of isolated transposon mutant DNA with restriction enzymes and ligation into a cloning vector such as pBluescript. SK II (Giles et al., 2015).

After sequencing, most commonly by Illumina, and mapping the sequencing reads to the genome, the disrupted genes are identified (Figure 1C). Fitness scores are assigned to those mutants based on their abundance compared to an untreated condition. To facilitate the ease of comparison across mutagenesis studies, fitness values are normalized to insertions in neutral sites; pseudogenes or those located in known intergenic regions where insertion is shown to cause no growth defect (Geisinger et al., 2019). The importance of Tn-seq has been demonstrated in a number of studies. It has been used to elucidate many pathways including the morphological switch from opaque to translucent corresponding to virulent and avirulent phenotypes in the American wound isolate AB5075 (Tipton et al., 2017; Chin et al., 2018) as well as the role of RelA in the same strain (Pérez-Varela et al., 2020).

### TraDIS and TraDISort

Transposon Directed Insertion Sequencing (TraDIS) (Langridge et al., 2009) is a technique that is highly similar to that of Tn-seq but differs slightly in sample preparation prior to sequencing (Figure 1B). The DNA is randomly cleaved resulting in variable sized fragments and during sequencing library preparation, an additional round of PCR is performed using a transposon specific primer and a sequencing specific adapter primer (Langridge et al., 2009; Barquist et al., 2016). Enrichment, such as this, allows for deeper sequencing coverage and thus higher confidence on transposition site but also introduces additional PCR bias as shorter templates are preferentially amplified over longer ones (Van Opijnen and Camilli, 2013). Utility of TraDIS was recently shown in a Vietnamese clinical isolate, where additional genes involved in colistin resistance such as those involved in membrane maintenance and regulation of transcription and translation were identified (Boinett et al., 2019). TraDIS has evaluated ATCC^®^ 17978^TM^ in respect to genes that are required for murine septicemia (Subashchandrabose et al., 2015). A study from the same group, generated a mutagenesis library in the clinical isolate AB0057 in order to mirror their initial study in ATCC^®^ 17978^TM^ (Crépin et al., 2018). Interestingly, in this study, to successfully select for the transposon insertion mutant pool, a kanamycin susceptible isogenic strain was created by deletion of *AB57_0288*, a gene that encodes aminoglycoside O-phosphotransferase. The mutagenesis was performed in this susceptible strain which also demonstrated similarities in murine virulence (Crépin et al., 2018). This highlights the challenges when working with MDR clinical strains as a susceptible parent strain is necessary to select for the transposon mutants. Due to this screen, a lytic transglycosylase, MltB, was revealed as essential in the murine infection model. With an elegant approach, Hassan et al. (2016) have identified a novel regulator for the efflux pump AvmA in AB5075 by taking advantage of the fluorescent property of the known efflux pump substrate, ethidium bromide, and combining it with TraDIS to create TraDISort. Using Fluorescence Associated Cell Sorting (FACS) to detect substrate fluorescence with TraDIS, TraDISort allowed for the selection of those mutants that had insertions in efflux pump encoding genes or their regulators based on their response to ethidium bromide. This method was validated with the identification of previously characterized efflux pumps, AdeABC and AdeIJK and although this is the only instance of TraDISort to date, provides insight into the power of TraDIS in combination with other sophisticated techniques in *A. baumannii.*

A very recent study in *Acinetobacter baylyi* demonstrated the power of Tn-seq to determine the importance of essential genes. Gallagher et al. (2020) harnessed the mutagenizing and sequencing aspects of Tn-seq coupled with the natural transformation efficiency of *Acinetobacter baylyi*. A transposon mutant library was generated using a Tn*5* transposon. The mutants’ DNA was pooled and transformed into wild-type *A. baylyi* then Tn-seq was performed over a specified time period. The speed by which the loss of DNA from those mutants whose transposon was integrated into an essential gene was detected *via* Tn-seq determined the criticality of the gene. In other words, the faster the depletion of mutant DNA from an essential gene integration, the more essential the gene to cellular functions. Although this study is limited to *A. baylyi*, it demonstrates further applications, similar to those shown by TraDISort, that can be coupled with transposon mutagenesis and their impact on the study of the organism as a whole.

## Genome Editing

### Homologous Recombination

**FIGURE 2:**
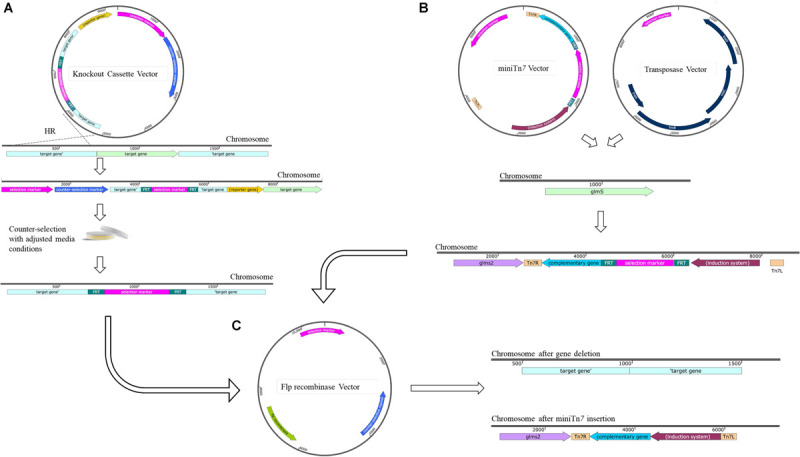
Current genetic techniques available for use in *A. baumannii* with common removal of selection marker. **(A)** Gene deletion via homologous recombination (HR) using suicide plasmid and A knockout cassette is designed with non-clinical antibiotic resistance gene of choice such as hygromycin or gentamicin flanked by Flp Recombinase Target (FRT) sites and homologous regions situated up and downstream of gene of interest. The knockout cassette can be then cloned into a suicide vector or maintained as a linear product. The use of counter-selection markers such as *sacB* in the presence of high quantities of sucrose enable curing of the vector. Unmarked deletions are then generated via the use of the Flp Recombinase acting on the engineered FRT sites. **(B)** Cloning and complementation with the minTn*7* system. In conjunction with the transposase, TnsABCE, integration of the miniTn*7* plasmid occurs at the neutral position upstream of the *glmS*2 gene. **(C)** Removal of the selection marker such as zeocin, apramycin, or hygromycin occurs via the Flp recombinase and is common between the mini-Tn*7* system and gene deletion via suicide plasmid. All maps were created using SnapGene software (from Insightful Science; available at snapgene.com). Not every feature of each plasmid is shown, only those relevant for selection, counter-selection and unique properties are displayed.

The genetic tools currently used for genome editing are based on harnessing existing molecular phenomenon. Homologous recombination, CRISPR and DNA repair mechanisms are such examples. One of the most common gene deletion strategies, previously and recently implemented with success in extensively drug resistant (XDR) strains of *A. baumannii*, uses a two-step homologous recombination approach (Figure 2A; Krasauskas et al., 2019; Xu Q. et al., 2019). Typically, this method involves the design of a knockout cassette that consists of an antibiotic resistance marker flanked by homologous regions of the target gene or adjacent. The cassette can be created using several methods such as PCR (Choi and Schweizer, 2005) or Gibson assembly (Gibson et al., 2009). There are two commonly used approaches to use the knock-out cassette, using suicide plasmids or using the linear DNA fragment.

### Gene Deletion Strategies Using Suicide Plasmids

The knockout cassette created can be cloned into a suicide knockout vector with selection (Table 1) and counter-selection markers (Table 2). Alternatively, the knock-out cassette can be assembled directly into the suicide vector using restriction digestion. However, with the advances in molecular techniques, this is the less desirable method due to limitations in unique restriction-digestion sites that can be used for cloning. The suicide vector containing the knock-out cassette is introduced into the host strain by electroporation, chemical transformation, or mating. The two-step allelic exchange, utilizing a non-replicative plasmid-based method follows (Hamad et al., 2009; Amin et al., 2013; Oh et al., 2015; Lee et al., 2018; Skerniškytė et al., 2019). Variations include those originally designed in *P. aeruginosa* and *Burkholderia* spp. The methods of Choi and Schweizer have been adapted for use in *A. baumannii* (De Silva and Kumar, 2018; De Silva et al., 2020) and includes the initial step of Splicing Overlap Extension (SOE), which introduces an antibiotic resistance gene flanked by Flp Recombinase Target (FRT) sites into the flanking homologous regions of the target gene to allow for selection of recombinants and antibiotic gene removal, respectively (Choi and Schweizer, 2005). Applications in clinical isolates using this method (Supplementary Table 1) have been successful depending on the type of selection and counter-selection markers used (Trebosc et al., 2016, 2019; Xu Q. et al., 2019).

**TABLE 1:** Summary of selection markers used in clinical isolates of *A. baumannii*.

**Selection marker**	**Type of marker**	**Genetic manipulation strategy**	**Strain**	**Selection use in recent reference(s)**
*hph*	Antibiotic	Transposon mutagenesis, complementation, unmarked gene deletion	AB5075 AbCAN2 MDR-ZJ06	Jacobs et al., 2014; Gallagher et al., 2015; Xu Q. et al., 2019; Lopez et al., 2020; Pérez-Varela et al., 2020
*ble*	Antibiotic	Complementation	HUMC1 AB031 LAC-4 ATCC^®^ 19606^T^ ATCC^®^ 17978^TM^ ACICU AYE	Luna et al., 2017; Ducas-Mowchun et al., 2019; Lucidi et al., 2019
*aac*(3)-IVa	Antibiotic	Complementation, CRISPR-based editing	ATCC^®^ 17978^TM^ ATCC^®^ 19606^T^ AB030 AB031 LAC-4 ABH6 XH386 AB5075	Ducas-Mowchun et al., 2019; Wang Y. et al., 2019; Pérez-Varela et al., 2020
*tet*	Antibiotic	Transposon mutagenesis	AB5075	Pérez-Varela et al., 2020
*kilA-telAB* or *tpm*	Toxic compound	Gene deletion, complementation	ATCC^®^ 17978^TM^ R2 DB BV26, BV94, BV173, BV175, BV185, BV186, BV187, BV189, BV190, BV191 HUMC1 Ab1 Ab2 Ab3	Amin et al., 2013; Trebosc et al., 2016, 2019; Luna et al., 2017; Charretier et al., 2018

*Use in specific strains has been noted. hph; hygromycin resistance, ble; zeocin resistance, aac(3)-IVa; apramycin resistance, kilA-telAB; tellurite resistance, tpm; tellurite resistance.*

**TABLE 2:** Summary of counter-selection markers used in clinical isolates of *A. baumannii*.

**Counter- selection marker**	**Type of marker**	**Strain**	**Use in recent studies**
*sacB*	Toxic compound in high concentrations	ATCC^®^ 17978^TM^ ATCC^®^ 19606^T^ BV26, BV94, BV173, BV175, BV185, BV186, BV187, BV189, BV190, BV191 AB030 AB031 LAC-4 ABH6 XH386	Amin et al., 2013; Oh et al., 2015; Ducas-Mowchun et al., 2019; Wang Y. et al., 2019; De Silva et al., 2020
*tdk*	Causes chain termination during DNA replication by incorporating 3*′*-azido-3*′*-deoxythymidine (AZT)	ATCC^®^ 17978^TM^ BV26 BV94 BV173 BV175 BV185 BV186 BV187 BV189 BV190 BV191	Trebosc et al., 2016, 2019
*lpxC*	Colistin susceptibility restored in Δ*lpxC* deletion mutant	ATCC^®^ 19606^T^	Lee et al., 2018

*Use in specific strains has been noted. sacB; sucrose toxicity, tdk; 3′-azido-3′-deoxythymidine (AZT) toxicity, lpxC; restoration of colistin susceptibility.*

The removal of the antibiotic cassette after the second homologous recombination event is highly desirable to avoid any possible polar effects (Figure 2C). Further, the presence of an antibiotic resistance cassette can interfere with phenotypic characterization such as susceptibility assays. Thus, the evaluation of a true gene deletion event cannot be accomplished, making the removal of the antibiotic resistance cassette necessary. This has been accomplished with remarkable success using the FLP recombinase system (Hoang et al., 1998). This system allows for the generation of unmarked deletions leaving behind the FRT scar sequences (De Silva and Kumar, 2018; Wang Y. et al., 2019). The introduction of additional bases in the form of the FRT site scars, have not been observed to have any pleiotropic effects. Several versions of the Flp recombinase-containing plasmids are now available. For example, those with a temperature-sensitive replicon (Choi et al., 2008) allow for greater ease of selection after the first recombination event. The modifications in the original Flp-recombinase containing vectors has allowed for their use in hyper-resistant clinical isolates of *A. baumannii* (Ducas-Mowchun et al., 2019).

### Gene Deletion Strategies Using Linear DNA Fragment

*A. baumannii* is well known to readily uptake exogenous DNA and this characteristic can be exploited for genetic manipulations (Domingues et al., 2019). One particular strategy utilizes the native DNA repair components, in particular the RecET recombinase system in combination with site-specific homologous recombination of a linear knockout cassette to achieve gene disruption in a single step (Tucker et al., 2014). The knockout cassette contains an antibiotic resistance selectable marker (Table 1), allowing for selection of cells with successful homologous recombination events. As with the previously mentioned approaches, removal of the antibiotic resistance marker can be performed by a Flp recombinase.

A recently developed single step homologous recombination technique is quite convenient (Trebosc et al., 2016) and allows for creation of scarless deletions via a single step method. The authors showed the versatility of this technique by deleting *adeR* (the gene encoding the response regulator that controls the expression of AdeABC efflux pump in tandem with the sensor kinase AdeS) in 10 different clinical strains isolated from various geographical sources (Trebosc et al., 2016). Scarless deletions also allow for the creation of multiple gene deletions in the same strain as the same selection marker can be reused. Again shown by further deleting *pmrA, eptA-1, eptA-2, eptA-3* in the same strains (Trebosc et al., 2019). Absence of antibiotic resistance gene in the knockout cassette excludes requirement to use the Flp recombinase making this approach scarless.

**FIGURE 3:**
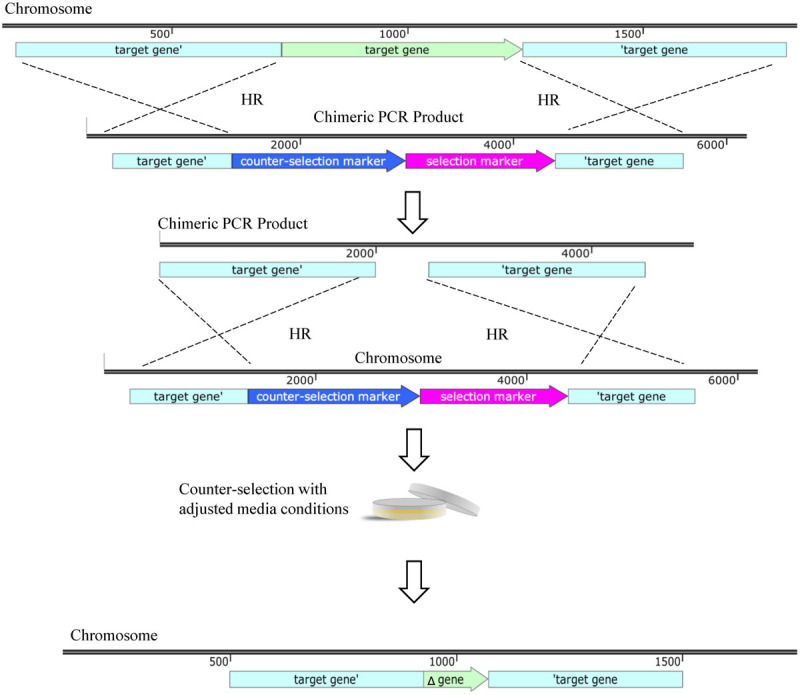
Linear DNA deletion technique. Gene deletion via homologous recombination (HR) using linear knockout cassette. A chimeric PCR product is designed with a selection marker such as apramycin resistance and a counter-selection marker such as *sacB* flanked by regions of homology to those surrounding the target gene. HR allows for disruption of target gene. A second chimeric PCR product is generated with homologous regions to the target gene and counter-selection allows for the second recombination event to select for removal of the selection and counter-selection markers leaving a scarless deletion. All maps were created using SnapGene software (from Insightful Science; available at snapgene.com). Not every feature of each plasmid is shown, only those relevant for selection, counter-selection and unique properties are displayed.

Of late, a two-step linear chimeric PCR product deletion method was validated via the deletion of the mobile genetic resistance element AbaR (Godeux et al., 2020). A PCR product is synthesized with *aacC4* and *sacB* genes flanked by regions homologous to those of the target gene (Figure 3). Transformation of such PCR chimera allows the first recombination to result in an interruption of the gene of interest with the apramycin resistance cassette and *sacB*. Cells after this first recombination event are selected on apramycin. To obtain a markerless deletion, an additional PCR product is generated with similar homologous flanking regions. Successful mutants are selected based on apramycin susceptibility and sucrose resistance. Strains AB5075 and AYE were subjected to this strategy and their AbaR resistance islands deleted as shown by reduced susceptibility of AYE to aminoglycosides, tetracycline and sulfonamides due to the loss of resistance island, similar to previous findings as a result of the loss of AbaR (Kochar et al., 2012). This strategy provides the advantage of a markerless, scarless deletion and is sensitive enough to produce single base-pair mutations (Godeux et al., 2020).

## CRISPR

### Immunological Role

Bacteria are constantly preyed upon by bacteriophages and therefore have evolved multiple antiviral strategies. Antiviral immunity of bacteria and archaea consists of a combination of innate and adaptive antiviral defense mechanisms. The innate defense mechanisms include receptor masking (Makarova et al., 2011, 2013; Samson et al., 2013), restriction–modification systems (Tock and Dryden, 2005), bacteriophage exclusion (BREX) (Goldfarb et al., 2015), Argonaute (Ago) family facilitated DNA interference (Swarts et al., 2014), and abortive infection (Chopin et al., 2005). Adaptive immunity is imparted through a Clustered Regularly Interspaced Short Palindromic Repeats (CRISPR) and associated Cas system. CRISPR-Cas is a common antiviral immunity system found in 45% of bacteria (Grissa et al., 2007). In addition to *cas* genes, a series of direct repeats and spacers termed as CRISPR arrays are responsible for target specificity. These spacers are derived from predatory viruses that infected the bacterium previously, thereby imparting immune memory (Bolotin et al., 2005; Mojica et al., 2005; Barrangou, 2015). By sequence specific targeting of the foreign (viral, plasmid) genetic material, the CRISPR-Cas system protects against mobile genetic elements (MGEs), thereby defending against viral predation (Barrangou et al., 2007; Brouns et al., 2008; Marraffini and Sontheimer, 2008; Hale et al., 2009; Garneau et al., 2010; Terns and Terns, 2011). The complexity of the CRISPR-Cas system warranted a comprehensive classification of the system. This was achieved by basing the classification on phylogenetic analysis of conserved Cas proteins, type and number of *cas* genes and gene arrangements (Makarova and Koonin, 2015). To date, four types and 12 subtypes (excludes six variant subtypes) of CRISPR-Cas system have been reported.

**FIGURE 4:**
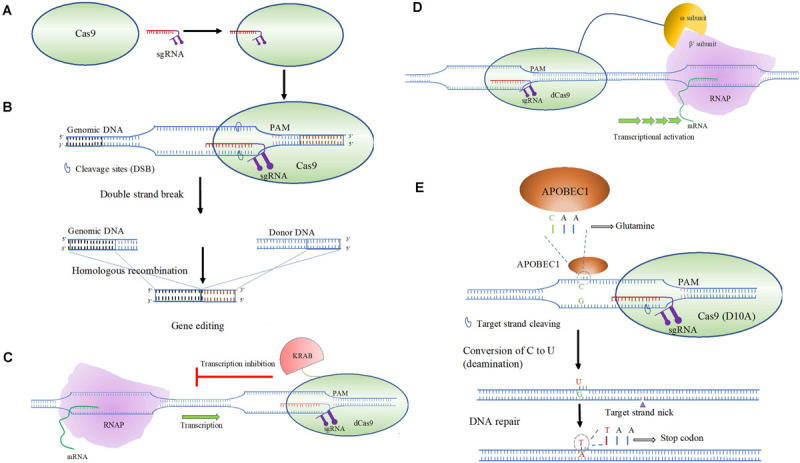
An overview of CRISPR-Cas9-based gene editing in bacteria. **(A)** Cas9 or a dCas9 binds to sgRNA that leads to an RNA-guided binding of the target DNA. The dCas9 may be assisted with transcriptional effectors (activator or an inhibitor). **(B)** Gene editing undertaken by Cas9-sgRNA target binding, double strand break and subsequent HR repair. **(C)** CRISPRi based targeted transcriptional inhibition through Cas9 fused to KRAB transcriptional inhibitor. **(D)** Activation of transcription driven by ω subunit of RNAP fused to dCas9, enabling RNAP recruitment through binding to the β’ subunit of RNAP. **(E)** Targeted base-editing performed by APOBEC1 fused to Cas9 (D10A), converting a triplet codon for glutamine to a stop codon.

The target recognition by a CRISPR-Cas system depend on the Cas protein binding Protospacer Adjacent Motif (PAM), leading to unwinding of the adjacent dsDNA helix (Sternberg et al., 2014; Redding et al., 2015; Jones et al., 2017). The RNA-directed aspect of the CRISPR-Cas is dependent on two RNAs, the CRISPR RNA (crRNA) and a *trans*-acting RNA (tracrRNA). The tracrRNA, which imparts partial complementarity, and the crRNA, can be combined to form a single guide RNA (sgRNA), that performs DNA target recognition and recruits Cas nuclease needed to perform genome editing (Figure 4A; Gasiunas et al., 2012; Jinek et al., 2012). The substrate specificity and the simplicity of the system that includes designing sgRNA makes CRSIPR an obvious choice for genetic manipulation. Following unwinding of the dsDNA, the single guide RNA (sgRNA) hybridizes to form a R-loop structure. This allows recognition of seed sequence near PAM elements enabling complementarity with the crRNA spacer and the CRISPR-Cas system to execute its effect (Figure 4B; Szczelkun et al., 2014; Rutkauskas et al., 2015; Jones et al., 2017; Xue et al., 2017).

### Utility in Genome Editing

In addition to creating specific gene knockouts, adaptation of the CRISPR-Cas system is implemented to perform interference or activation, termed CRISPRi and CRISPRa, respectively, enabling transcriptional control (Qi et al., 2013). Transcriptional repression or knockdown *via* CRISPRi is an alternative to mRNA targeting RNAi and differs from the later as it may block either transcriptional initiation or elongation (Figure 4C). This system uses a deactivated nicking endonuclease Cas9 (dCas9) for a targeted RNA-directed transcriptional control. dCas9 may need additional helper proteins or effector proteins that allows higher degree of transcriptional repression. As shown in Figure 4C, notable effector proteins include KRAB (Krüppel-associated box) domain of Kox1 and the CS (chromoshadow) (Qi and Lo, 2017). Bacterial gene knockdown using CRISPRi has been investigated in Gram-negative *E. coli* (Jiang et al., 2013; Li et al., 2016) and Gram-positive *Bacillus subtilis* (Peters et al., 2016). This approach is tunable and can be modified to perform both moderate and high degree of over-expression and knockdown by using inducible promoters (Li et al., 2016). Alternatively, CRISPRa is a targeted gene activation approach, where a transcriptional activator domain or a subunit of RNAP could be fused to dCas9 augmenting rate or initiation of transcription through RNAP recruitment (Figure 4D; Dong et al., 2018). Use of omega (ω) subunit of RNAP has shown to be an effective transcriptional activator in *E. coli* (Dove and Hochschild, 1998; Bikard et al., 2013).

### Diversity of CRISPR Systems

The utility of CRISPR-Cas9 system (Figure 4) has been demonstrated in Gram-negative bacteria including *E. coli*, *P. aeruginosa*, and *Burkholderia* spp. (Jiang et al., 2015; Chen et al., 2018; Hogan et al., 2019). Previous studies showed instances where mismatch and/or partial match of sgRNA to target DNA is tolerated often leading to off-target effects (Graham and Root, 2015; Lee et al., 2016). Although shown in mammalian cell lines, it is possible that in-frame mutations affect the CRISPR based knock-out efficiency. The reduction of in-frame mutation was addressed by the authors by developing a microhomology prediction thereby improving knock-out efficiency (Bae et al., 2014). A large volume of data on genome editing is currently available. To assist a CRISPR based gene editing approach as a method of genome editing, by improving both efficiency and accuracy, we rely on multiple computational tools, which are covered extensively in a review published previously (Chuai et al., 2017). The scope of the use of computational tools encompasses *in silico* sgRNA design to enhance target specificity, CRISPR based sgRNA off-target profiles and analyzing outcomes of CRISPR experiments. These use posterior NGS to characterize insertions, deletions, and homologous recombination at the intended target sites. Even with the advancement of *in silico* methods in predicting off-target effects, the measured process recently proposed combining both *in silico* and experimental approaches (Hendel et al., 2015; Lee et al., 2016). At the core of these approaches remain NGS and subsequent data analysis to identify functional cleavage sites, in combination with genome wide techniques including double-strand break (DSB) capture and protein binding site capture (Lee et al., 2016). Another study (Karlapudi et al., 2018) using *A. baumannii* strain M2, evaluated the application of web-based tools (CHOPCHOP, CRISPR-ERA, CCTop and E-CRISP) for *in silico* sgRNA design. Individual web-based tools consider one or multiple relevant factors that includes location of (PAM), sequence alignment scores attributed to GC content and exon position thereby predicting on-target efficacy.

### Exogenous CRISPR Systems in *A. baumannii*

In order to adapt a CRISPR based system to be used in *A. baumannii*, several modifications to the system were made and tested. Based on the strategy previously outlined by Hsu et al. (2013), the authors (Wang Y. et al., 2019) constructed a plasmid containing expression cassettes for Cas9 nuclease from *Streptococcus pyogenes* and the corresponding sgRNA (Hsu et al., 2013). In addition, to enhance the recombination efficiency of weak homologous recombination based DSB repair found in *A. baumannii*, authors developed an exogenous recombination system, that involves a two-plasmid system with each plasmid capable of replicating in both *E. coli* and *A. baumannii* (Fürste et al., 1986). The selection of RecAb from the *A. baumannii* IS-123 strain (Tucker et al., 2014) over recombinases from other species resulted in > 10-fold higher efficiency, reflected in the higher number of colonies selected for during a targeted deletion of the regulator *oxyR*. The amount and length of the repair template was also optimized, which greatly improved the editing efficiency. The authors also tested genome editing efficiency that is demonstrated in lab strains ATCC^®^ 17978^TM^, and ATCC^®^ 19606^T^ and in one clinical isolate *A. baumannii* ABH2. The reason for the use of multiple strains was the expected variation in efficiency of CRISPR-Cas based genome editing owing to differences in genomic background.

To investigate amino acid residues in the active site of OxyR that are critical for H_2_O_2_ sensing, point mutations were introduced in ABH2 using the exogenous CRISPR-Cas system (Wang Y. et al., 2019). By inserting an artificial spacer to replace the native spacer and then introducing a point mutation during restoration of the native spacer, 13 mutant strains were generated. Three of these mutations included conserved non-critical amino acids T104, H201, and R270 found in OxyR of other bacteria. These strains as expected did not show any deficiency with respect to H_2_O_2_ sensitivity. A known residue, C202, and three new residues, E130, S133, and S226 were determined to be critical for OxyR function. In *A. baumannii*, many acquired resistance genes are associated with mobile elements (plasmids and transposons), and undesired recombination events or the loss of a plasmid encoding CRISPR elements may occur when performing CRISPR-Cas based gene editing (Wang et al., 2018). An alternative approach is a cytidine base-editing system of targeted C to T conversion to achieve gene disruption. This method is independent of DSB and the need of a donor template. A C to T conversion can change a codon, for example CAA or CAG to a stop codon (TAA or TAG) (Figure 4E). A single plasmid system pBECAb-apr was constructed containing expression cassettes for a sgRNA and a fusion protein. One of the proteins in the fusion was a APOBEC1 deaminase that catalyzes the conversion of C to U in the displaced DNA strands. The other, Cas9(D10A) nickase cleaves the non-edited DNA strand. DNA repair or replication converts U:G originated from C:G to T:A. The functionality of the cytidine base-editing system was confirmed in *A. baumannii* ATCC^®^ 17978^TM^.

As an exogenous CRISPR-Cas system may fail in some species, functional endogenous CRISPR-*cas* in many bacteria had been used for successful gene editing. In *Haloferax volcanii*, an archaeon, endogenous CRISPR-Cas system was used for gene repression through CRISPRi (Stachler and Marchfelder, 2016). In *Clostridium tyrobutyricum*, a Gram-positive bacterium, single and multi-gene deletions were successfully realized using the endogenous type I (subtype B) CRISPR-Cas system. In another study, an editing efficiency of 100% was achieved for the CRISPR-cas based gene deletions to develop strains for high-level butanol production (Zhang et al., 2018). A 100% efficiency for CRISPR-Cas based gene deletion was reported for a 643 bp deletion, and lower efficiencies for insertion and single nucleotide substitution (at 36 and 19% efficiency, respectively) performed in *Lactobacillus crispatus*, a Gram-positive commensal bacteria found in vagina and intestinal tracts (Hidalgo-Cantabrana et al., 2019). The researchers used the endogenous type I (subtype E) CRISPR-Cas system for the three gene editing approaches reported here. In *E. coli*, two studies reported the use of endogenous type I CRISPR-Cas system to modulate metabolic pathways *via* gene editing and CRISPRi, using Cas3 and dCas3 proteins, respectively (Chang et al., 2016; Tarasava et al., 2018). In *S. aureus*, type III (subtype A) CRISPR-Cas system has been used to achieve gene deletions and insertions (Guan et al., 2017). The above examples of successful use of endogenous CRISPR-Cas systems in archaea, Gram-positives and Gram-negatives highlights the possibilities of their use in other bacteria.

### Endogenous CRISPR Systems in *A. baumannii*

Analysis of CRISPR database (CRISPRdb) showed two CRISPR-*cas* in *Acinetobacter* spp. The first system is discovered in the genome of clinical isolates *A. baumannii* AYE, AB0057, and AB037 (Grissa et al., 2007; Hauck et al., 2012). Another study demonstrated the presence of G55 islands in two of the above three strains AB0057 and AYE, and an additional strain 4,190 (Di Nocera et al., 2011). These G55 island contains the CRISPR blocks flanked by a *cas* gene cluster. The second of the CRISPR-*cas* system in *Acinetobacter* spp. was found in *A. baumannii* type strain ATCC^®^ 19606^T^ and in *A. baylyi* ADP1. The large volume of sequencing data and available bioinformatics tools have permitted the identification of CRISPRs in various *A. baumannii* strains (Grissa et al., 2009). The spacer organization in CRISPR-*cas* have been used for comparison between strains. A pangenome study encompassing 2,500 genomes of *A. baumannii*, agreed to the presence of two CRISPR-Cas systems (Mangas et al., 2019). By constructing a new phylogeny of *Acinetobacter* based on presence or absence of 14 common genes, they divided the genomes in to two groups. The first group, sharing fewer common genes, showed gene enrichment for maintaining CRISPR elements and rarity in the presence of plasmids. The authors in addition to confirming type 1 system showed the presence of type IV CRISPR-*cas* in *A. baumannii* as previously suggested. The relationship between CRISPR-*cas* and virulence genes have been discussed in a study (Louwen et al., 2014). With the success of endogenous CRISPR-Cas system including type I in genome editing in multiple bacteria strains, the same may be explored in *A. baumannii.* In addition, useful information would be obtained by better understanding of the relationship of CRISPR-Cas systems in *A. baumannii* with the virulence and pathogenesis.

Here we have summarized the use of both exogenous and recently explored endogenous CRISPR-cas system in gene manipulation. Although there are only a handful examples of using the CRISPR-Cas system in *A. baumannii*, there is certainly a tremendous potential for this technique to be used in this organism.

## Complementation and Cloning Approaches

Several vectors are now available for cloning in *A. baumannii* (Supplementary Table 3). There are two common approaches for complementation that include chromosomal or extra-chromosomal. Extra-chromosomal cloning systems consists of plasmids, which have been mainstay of the cloning strategies historically. However, chromosomal insertion systems are increasingly becoming staple for cloning approaches, particularly since these systems allow expression of complemented genes at levels that mimic what is seen in nature and also because such systems do not require antibiotic selection facilitating *in vivo* studies.

### Single Copy Insertion Systems

The most widely used mini-Tn*7* system was originally developed for chromosomal insertions in *P. aeruginosa* (Choi and Schweizer, 2005). This system has been modified for use in *A*. *baumannii* (Figure 2B). With the help of exogenously supplied transposase, single copy Tn*7* transposition occurs at a very specific *att*Tn*7* site within the genome. In *A. baumannii* this is located in the intergenic region 24 base pairs upstream of the *glmS2* gene (Kumar et al., 2010). Based on its conservation across species, the resulting *att*Tn*7* site is presumed to be a neutral site within the chromosome (Choi and Schweizer, 2006; Choi et al., 2006, 2008; Kumar et al., 2006). There are a variety of iterations of this system including various selection markers, and induction parameters (Supplementary Table 3). Versatility of this system has been shown by its successful use in AB5075 (Pérez-Varela et al., 2020; Williams et al., 2020) as well in the clinical isolates AB030, AB031, and LAC-4 with the chromosomal insertion of constitutive fluorescent markers (Supplementary Table 4) including mTurquoise, RubyRed, mCherry, and sfGFP (Ducas-Mowchun et al., 2019), as well as complementation of deletion mutants in ATCC^®^ 17978^TM^ using the gentamicin selectable marker (De Silva et al., 2020). The antibiotic selection marker is removed via the Flp recombinase method described above (Figure 2C) using pFLP2 derivatives (Supplementary Table 2). Insertional complementation provides the benefit of maintaining single copy insertion of the gene of interest, allowing for expression at physiological levels and maintenance of complementation on selectable -free media reducing the risk of mutations via antibiotic stress. While Tn*7*-based systems have been used successfully in various strains of *A. baumannii*, it should be noted that *A. baumannii* strains may contain more than one copy of *glmS* (Kumar et al., 2010). The insertion of Tn*7* element has always been shown to occur downstream of *glmS2*, but researchers should ideally confirm the insertion site when using this system with a new strain of *A. baumannii*. As with any transposition, immunity is also a concern using this system which prevents multiple cloning strategies in a single strain. Similar to the removal of the selectable marker during gene deletion, the FRT scar is left behind in the newly generated strain.

Alternatively, the use of endogenous site-specific recombination systems also show success in removal of antibiotic markers. The XerC/D tyrosine recombinases recognize adjacent *dif* sites within the newly replicated chromosome and aid in the separation of daughter chromosomes during replication. Many *dif* sites have been identified on plasmids within clinical isolates, flanking *bla*_OXA__–__58_ and contribute to the mobility of the gene (Cameranesi et al., 2018). This system has been modified for use in removal of antibiotic cassettes in reporter strains of clinical *A. baumannii* isolates (Supplementary Table 2). An autoluminescent strain was generated using the Tn*7* system and removal of the cassette performed via the XerC/D recombineering strategy (Jiang et al., 2019). Removing the selectable marker through this approach allows for its removal while leaving no genetic scar.

### Plasmid-Based Systems

Plasmid-based systems continue to be utilized by various groups, primarily due to the ease in their use. A number of plasmids used in *A. baumannii* are described in the review by Biswas (2015). However, since then, some useful plasmids have been designed for use in *A. baumannii*. We highlight the evolution of the newest systems below.

#### pVRL System

Extra-chromosomal plasmid-based complementation techniques have also been fine-tuned for *A. baumannii.* Provide complementation of *gnaA* in the MDR clinical isolate MDR-ZJ06 (Zhou et al., 2011), using a pET28a based *E. coli* shuttle vector pYMAb2 (Supplementary Table 3), that was modified to allow for hygromycin selection. Restoration of the phenotype upon complementation was observed except during the virulence assay with the wax worm model, *Galleria mellonella* as maintenance of the plasmid vector *in vivo* can be unreliable Xu Q. et al. (2019). Thus, underscoring the utility of genome insertion systems for *in vivo* experiments. Nevertheless, the pVRL system has been shown to function in *A. baumannii* as well as in *A. baylyi* (Lucidi et al., 2018). There are a variety of shuttle vectors in this family. pVRL1 and pVRL2 provide blue/white selection abilities for selection in *E. coli* as well as a gentamicin cassette for selection in *A. baumannii* (Lucidi et al., 2018).

#### pLVP System

A useful set of high copy plasmids were developed by Lucidi et al. (2019) that include reporter systems compatible in the clinical strains ACICU and AYE. Using the *uvr*-dependent DNA repair pathway as a proof of concept, activation of its promoter was evaluated in ATCC^®^ 19606^T^ by cloning the promoter into pLVP1Z, pLVP2Z, and pLVP3Z (Supplementary Table 4). Luminescence, β-galactosidase activity, and fluorescence were detected from all promoter fusions during exposure to UV, demonstrating the successful generation of a reporter strain in ATCC^®^ 19606^T^. This system was further validated with the use of the ethanol inducible promoters in ATCC^®^ 17978^TM^, ATCC^®^ 19606^T^ and AYE using pLVP1Z and the promoter regions of the ethanol dehydrogenase encoding *adhP* and the aldehyde dehydrogenase *yahK*. Again, the *lux* promoter fusion produced luminescence in a dose dependent manner when exposed to ethanol (Lucidi et al., 2019). In a final evaluation, iron regulation involving the Fur-Fe repressor complex and the *basA* promoter was evaluated in ATCC^®^ 19606^T^ using all three vectors. Reporter strains were exposed to increasing concentrations of FeCl_3_ in minimal media and a decrease in luminescence, β-galactosidase activity and fluorescence were observed. With the use of pLVP1Z::P*_*basA*_*, under iron limiting conditions, Lucidi et al. demonstrated that in the animal model, *G. mellonella*, luminescence was detected and decreased over time as iron was scavenged from the host. This highlights the utility of these vectors for *in vivo* experiments. As with other extra-chromosomal cloning systems, these vectors must be selected for, which poses challenges as antibiotics modify cellular functions. The pLVP system, however, has been shown to be stable over 40 generations without an antibiotic selection and therefore is still of great value (Lucidi et al., 2019).

## Selection Markers for Clinical Isolates

### Non-clinical Antibiotic Markers

One major obstacle when working with clinical isolates of *A. baumannii* is that most, if not all are MDR or even XDR. This presents challenges when selecting for genetically manipulated mutants of interest as historically, those mutants are selected by an antibiotic resistance marker that differentiates them from wild-type cells. For this approach to work, high concentrations of many commonly used antibiotics are used that may introduce additional mutations. Alternatively, for manipulations of clinical isolates, non-clinical antibiotics and toxic compounds are used such as hygromycin, zeocin, apramycin, and tellurite. Novel vectors must be developed with these less common selection markers to increase the success of studies performed in MDR/XDR isolates.

Both hygromycin and apramycin are aminoglycoside antibiotics whose use is restricted to veterinary medicine making it appealing for application in *A. baumannii* isolates from clinical sources (Kotra et al., 2000). Hygromycin-specific resistance is encoded by a single gene, *hph* (Gritz and Davies, 1983) highlighting the ease of use in cloning into new vectors. This marker has been used for transposon mutant selection (Jacobs et al., 2014; Gallagher et al., 2015) and complementation experiments (Pérez-Varela et al., 2020) in AB5075. It has also been implemented successfully in AbCAN2 (Lopez et al., 2020), as a selection marker for the Flp recombinase expressing vector (Table 1).

Alternatively, apramycin resistance is conferred by 3-N-acetyltransferase type IV gene, *aac(3)-IVa* (Paget and Davies, 1996) that also provides a wide range of resistance to other aminoglycosides (Table 1). Successful genetic manipulations include complementation with the mini-Tn*7* based integration system in XDR isolates (Ducas-Mowchun et al., 2019), and AB5075 (Pérez-Varela et al., 2020) as well as the CRISPR gene editing maintenance of the Cas expressing vector in clinical isolates ABH6 and XH386, and type-strains ATCC^®^ 17978^TM^ and ATCC^®^ 19606^T^ (Wang Y. et al., 2019). Zeocin is an antibiotic that causes cell damage by intercalation into dsDNA causing lethal double-stranded breaks (Gatignol et al., 1988) and is not in clinical use. Therefore, most clinical isolates of *A. baumannii* are susceptible to zeocin. Utility of zeocin as a selection marker has been shown in a number of clinical isolates from various countries (Luna et al., 2017; Ducas-Mowchun et al., 2019; Lucidi et al., 2019; Table 1).

### Non-antibiotic Markers

Non-antibiotic markers are beneficial because resistance is less readily acquired as these are typically absent from mobile genetic elements. Amin et al. (2013) and Luna et al. (2017) made use of the tellurite resistance cassette and the *xylE* reporter gene to select for gene deletions in MDR isolates DB, R2, and HUMC1, respectively (Supplementary Table 2). Tellurite oxides are thought to cause damage to cells via oxidative stress and therefore exhibit activity in a wide range of bacterial species and cells expressing Tel^R^-marker also show black pigmentation in Tel-supplemented medium which can further aid in the selection (Sanchez-Romero et al., 1998). The pABBR-TelR (Supplementary Table 3) construct was modified for use in clinical isolates to study the role of alleles of *pmrAB* in clinical colistin heteroresistance (Charretier et al., 2018). In conjunction with the Tel^R^ gene, *tpm* (Trebosc et al., 2016), or the *kilA-telAB* operon (Amin et al., 2013), the *xylE* reporter gene can be used to visualize color change in a colony to signify those with successful first cross-over events. The *xylE* gene product is a dioxygenase that hydrolyzes pyrocatechol to produce a yellow color, visible unaided, allowing for simple segregation of desired mutants (Ingram et al., 1989). Gene deletion in clinical isolates from Switzerland, United States, Turkey, Greece, Mexico, Spain and China using these selection markers was successful (Trebosc et al., 2016, 2019).

## Counter-Selection Markers for Clinical Isolates

In an ideal situation, genetic modifications would be free of selection markers used during strain generation, ensuring no interference in downstream assays. In order to remove plasmids during genetic manipulations, a counter-selection marker allows for curing of the vector. Stringency of conditions for counter-selection is challenging and, in many cases, must be optimized on a strain by strain basis (Khetrapal et al., 2015). There are only two common counter-selection systems (*sacB* and *tdk*) currently in use for widespread applications in *A. baumannii*. These are methods based on lethality in the presence of high amounts of sucrose or 3′-azido-3′-deoxythymidine (AZT). The limited number of counter-selection markers currently available for use in *A. baumannii* necessitates that more options are needed particularly since the efficiency of existing markers can be quite variable from strain to strain.

### Counter-Selection With *sacB* and *tdk*

In the case of sucrose counter-selection (Table 2), a vector is engineered with the *sacB* gene, originally isolated from *B. subtilis*, which encodes a levanosucrase. This enzyme performs the transfructorylation reaction of sucrose into levan in high sucrose environments and the buildup of this metabolite is toxic to many Gram-negative organisms including *A. baumannii* (Reyrat et al., 1998). This counter-selection marker has been used mainly to select for the second recombination event during homologous recombination-type strategies (Amin et al., 2013; Oh et al., 2015; De Silva et al., 2020) or to remove the Flp recombinase expressing vector (Ducas-Mowchun et al., 2019; De Silva et al., 2020) in type strains but also with some success in clinical isolates such as AB030 and AB031. *sacB* is by far the most commonly used counter-selection marker. It has recently been shown useful in curing of CRISPR based plasmids after gene editing in ABH6 and XH386 (Wang Y. et al., 2019). Efficiency of this system can vary from strain to strain and thus standardizing the growth conditions may be required. These include optimizing the sucrose concentration, incubation temperature, and/or the growth medium.

However, it has been observed that *sacB* can result in toxicity and thus select for mutations in *sacB* resulting in the maintenance of the vector in cells under counter-selection conditions (Trebosc et al., 2016). As a result, *tdk* and its cognate small molecule inhibitor AZT (Table 2) has been used as an alternate counterselection marker (Trebosc et al., 2016). In *E. coli*, the gene *tdk* encodes a thymidine kinase which is a key enzyme in the phosphorylation of thymine during nucleotide synthesis. With the introduction of the thymidine analog AZT, Tdk incorporates this dideoxynucleotide during DNA replication forcing chain termination, a lethal event. This counter-selection marker has been shown be quite effective and does not appear to select for mutations in *tdk* (Trebosc et al., 2016, 2019).

### Counter-Selection With *lpxC*

Lee et al. (2018), introduce a variation on the previous counter-selection practices. It is known that the mechanism for colistin resistance results from loss of lipid A (Moffatt et al., 2010), modification of LOS (Beceiro et al., 2011) or mutations in the two-component system *pmrAB* (Charretier et al., 2018). Linking the gain of lipid A with restored colistin susceptibility, *lpxC* was cloned into a suicide vector (Supplementary Table 2). Generating a Δ*lpxC* mutant strain allowed for loss of colistin susceptibility and therefore a method for counter-selection. In order to maintain survival on colistin the mutant strain cures the vector after homologous recombination occurs with the gene of interest. However, the major limitation of the method is that it can only function in the Δ*lpxC* background, limiting the widespread application of this marker. Colistin, being the last resort antibiotic for *A. baumannii* infections, is also not an ideal antibiotic to be used for selection in laboratory conditions. Although creative, this method may not be of much use for manipulating clinical isolates of *A. baumannii*.

## Other Common Counter-Selection Systems

Since there are only a limited number of counter-selection markers that have been used in *A. baumannii*, it is worthwhile investigating other options. A variety of counter-selection markers have been engineered for use in other bacterial species, described below in brief. Some of these counter-selection markers may be useful in *A. baumannii*.

### *rpsL* and *tetAR*

*rpsL* encodes the ribosomal subunit S12, which is the target of the antibiotic streptomycin. Presence of this gene increases the susceptibility of those cells to streptomycin and selective pressure ensure loss of the vector containing *rpsL* and success has been seen in *E. coli, Bortedella pertussis, Mycobacterium smegmatis* (Reyrat et al., 1998) and recently in *Corynebacterium glutamicium* (Wang T. et al., 2019) *and Borrelia burgdorferi* (Drecktrah et al., 2010). Constant use of this marker selects for streptomycin resistant mutants which if used as a selection marker would be a detriment but further, its use in *A. baumannii* is plausible as clinical strains tend to be intrinsically resistant to streptomycin (Magnet et al., 2001). Similarly, the use of *tetAR*, confers resistance to tetracycline but vulnerability to fusaric acid. However, these methods can unfortunately select for tetracycline resistant mutants (Reyrat et al., 1998). With the MDR phenotypes of clinical isolates, antibiotic based counter-selection markers present challenges in manipulations of clinically relevant *A. baumannii*.

### mazF

Recently, the toxin component of a toxin-anti-toxin system from *E. coli* was used as counter-selection in the Lactic Acid Bacteria (LAB), *Lactobacillus plantarum* and *Enterococcus mundtii* (Van Zyl et al., 2019). Expression of *mazF* results in cell death by the cleavage of mRNA at ACA sites. Cloned with the nisin inducible promoter, this counter-selectable marker is under tight regulatory control to prevent leaky expression and undesired lethality. Genetic manipulation using *mazF* has been demonstrated in *B. subtilis* (Zhang et al., 2006), *Clostridia* spp. (Joseph et al., 2018), and the yeast *Pichia pastoris* (Yang et al., 2009). Since the nisin inducible promoter’s efficacy in Gram-negatives has also yet to be determined, the utility of this system in *A. baumannii* remains to be seen.

### *pheS* Variant

In Gram-positives, a common counter-selection marker is *pheS*^∗^. Encoding for a variant of the subunit of the phenylalanine tRNA transferase, this allows for the loading of para-chlorophenylalanine (PCPA) instead of only phenylalanine onto the tRNA^Phe^ (Ibba et al., 1994). In the presence of PCPA, this results in misfolding of proteins and eventually cell death. Successful counter-selection has been achieved in Gram-positives such as *Streptococcus mutans* (Xie et al., 2011) and *Enterococcus faecalis* (Van Zyl et al., 2019), more recently in *Staphylococcus aureus* (Schuster et al., 2019) and Gram-negative organisms such as *Burkholderia pseudomallei* (Barrett et al., 2008). Currently *pheS*^∗^ has not been manipulated for use in *A. baumannii* nor *P. aeruginosa* but considering its success in *B. pseudomallei*, could be an addition to the arsenal of counter-selection markers for *Acinetobacter* spp. A “super lethal” PheS in *E. coli* has been established (Miyazaki, 2015) which may also lend itself for use in *A. baumannii.* However, the utility of PheS in *A. baumannii* remains to be explored.

## Discussion

Considering the evolution of *A. baumannii* as a major Gram-negative pathogen of concern in hospitals around the world, it is critical that research stay current and cutting edge. To evaluate emerging clinical isolates, it is vital to develop and maintain methods for their genetic manipulation. Such methods allow for investigation into mechanisms of virulence and resistance, allowing for further improvements to current treatment options. As the majority of the work in *A. baumannii* has been carried out in the laboratory strains ATCC^®^ 17978^TM^ and ATCC^®^ 19606^T^, applying this knowledge and these techniques to more recently isolated clinical strains is important. It has also been noted that these strains may not represent the characteristics that they once had or those strains in the current clinical environment. This is in part because they lack the MDR/XDR and highly virulent phenotypes common among current clinical isolates but also have evolved a distinct resistance profile from their origin (Harding et al., 2018). This has resulted in research groups using modern clinical strains as type-strains for their studies. One such example is AB5075 (Supplementary Table 1). It is a MDR strain, that was first isolated from the United States military health care system, and has been selected as a representative of clonal complex I as well for its increased virulence in a murine model (Jacobs et al., 2014). It is sensitive to hygromycin and tetracycline allowing for genetic manipulations with these antibiotic selectable markers and has been genetically modified extensively to study a multitude of mechanisms encompassing resistance, virulence and global regulation of cellular processes. In addition, this strain exhibits phase variation that has been attributed to virulence (Chin et al., 2018) and this phenomenon is being investigated in other isolates (Ahmad et al., 2019). Depending on the phase, phenotypic characteristics such as biofilm formation, antibiotic susceptibility, motility, and virulence differ suggesting that great care must be taken when working with this strain and others (Tipton et al., 2015).

In Europe, much work has been done on AYE (International Clone I) and ACICU (International Clone II, also known as European Clone II). AYE, a MDR strain endemic to France (Fournier et al., 2006) is susceptible to non-clinical antibiotics such as apramycin and has been manipulated successfully since its isolation in 2006 (Lucidi et al., 2018, 2019). Comparisons of deletions of *adeRS* and *adeB* have been made with strain AYE and S1 with differences in virulence, biofilm formation and antibiotic susceptibility (Richmond et al., 2016). The Italian isolate ACICU, also a MDR strain, has been studied extensively (Iacono et al., 2008) and has been used as a comparator for novel mutations in colistin resistance (Gerson et al., 2020) and as a reference for genomic assemblies (Yang-Yi Tan et al., 2013; Hamidian et al., 2019).

Additionally, AB030 and AB031 are clinical isolates from Canada representing highly virulent strains with contrasting resistance phenotypes. AB030 is highly virulent and XDR, whereas AB031 is highly virulent but less drug-resistant than AB030 (Fernando et al., 2013; Singh et al., 2020). Use of clinically relevant selection markers are critical in AB030 such as apramycin, hygromycin and zeocin whereas these are not required in AB031 as this strain is susceptible to classical markers such as gentamicin. This pair allow for the comparison of those systems that may contribute to resistance and virulence. AbCAN2 is also Canadian in origin and the T6SS has been heavily studied in this isolate (Di Venanzio et al., 2019; Lopez et al., 2020).

In *A. baumannii* research, it is imperative to be aware of distinctions between strains. Many comparisons have been performed on a wide selection of isolates revealing genomic and phenotypic differences in antibiotic resistance capabilities, virulence factors as well as clonal lineages (Fournier et al., 2006; Fernando et al., 2013; Casella et al., 2017; Cerezales et al., 2020; Liu et al., 2020; Singh et al., 2020). To aid in research efforts, the Multi-drug organism Repository and Surveillance Network (MRSN) has accumulated 3,500 *A. baumannii* isolates from around the world and have short-listed 100 strains that represent the genetic diversity and maximal phylogenetic distance (Galac et al., 2020). Significant effort must be dedicated to the study of the diverse nature of this pathogen. No matter the strain being studied, whether it be ATCC^®^ 17978^TM^, ATCC^®^ 19606^T^ or a more recent isolate, these variations must be carefully considered when comparing results.

The advancement of techniques for use in clinical strains has been aided by the use of non-clinical and non-antibiotic selection markers, both in deletion and complementation methods. Recent progress with the introduction of an *A. baumannii* adapted CRISPR-Cas system to edit and delete genes holds great promise (Wang Y. et al., 2019). CRISPR provides high specificity and allows for base editing and gene deletion in clinical isolates. Although it is relatively new and has only been demonstrated in two strains of different ST’s, it is a promising tool whose use can be expanded to include many clinical isolates.

Challenges with counter-selection remain at bay to date but should be explored to enhance future genetic techniques. Sucrose and AZT remain the gold standards in clinical isolates, but toxicity associated with *sacB* has already been noted (Trebosc et al., 2016). Promising lethal gene-cognate small molecule pairs have been identified in other species such as *pheS*^∗^/PCPA and induction of *mazF*. Active investigation into these counter-selection markers allows for an increase in quantity of systems to choose from. It also provides options for use in novel *A. baumannii* strains.

There have been significant efforts made to advance the ability to genetically manipulate *A. baumannii*, specifically clinical isolates. These advancements significantly help in the understanding of the current disease conditions better and further the ability to develop treatment options for such infections.

## Author Contributions

EMES, SD, and AK conceptualized and wrote the manuscript. All authors contributed to the article and approved the submitted version.

## Conflict of Interest

The authors declare that the research was conducted in the absence of any commercial or financial relationships that could be construed as a potential conflict of interest.
