# Supplementary material for: Recent Advances in Genetic Tools for Acinetobacter baumannii
Source: Front Genet. 2020 Dec 22;11:601380. doi: 10.3389/fgene.2020.601380 (PMC7783400; doi:10.3389/fgene.2020.601380)
Supplement: Supplementary file 1 [file Table_1.docx]

Supplementary Material

**Supplementary Table 1: Clinical and Laboratory Strains of *A. baumannii* with Successful Genetic Manipulations Conducted.**

| **Strain** | **Source** | **Country** | **Lineage** | **Accession Number** | **Reference** |
| --- | --- | --- | --- | --- | --- |
| ATCC17978 | Fatal meningitis | France (1951) | ST112  (Luo *et al*., 2012)  MLST77 | NC_009085.1 [NZ_CP018664.1](https://www.ncbi.nlm.nih.gov/nuccore/NZ_CP018664.1) NZ_CP053098.1 CP012004.1 | (Mussi *et al*., 2011) (Trebosc *et al*., 2016) |
| ATCC19606 | Urine | USA (Before 1949) | MLST52 | SRR10295884 | (Zhu *et al*., 2020) |
| AB030 | Bloodstream infection | Canada | ST758, International Clone V | NZ_CP009257.1 | (Fernando *et al*., 2013) (Singh *et al*., 2020) |
| AB031 | Bloodstream infection | Canada | International Clone V | NZ_[CP009256](https://mra.asm.org/lookup/external-ref?link_type=ncbi%3Anucleotide&access_num=CP009256&atom=%2Fga%2F2%2F5%2Fe01036-14.atom).1 | (Fernando *et al*., 2013) |
| LAC-4 | Nosocomial outbreak- reliably reproduces most relevant features of human pulmonary *A. baumannii* infection | Los Angeles, USA | ST10 | NZ_CP007712.1 | (Oh *et al*., 2015) |
| R2 | Urine sample- patient with malignant lymphoma and pneumonia | Japan (Teikyo University Hospital) | Non clonal | N/A | (Sato *et al*., 2018) |

| **Strain** | **Source** | **Country** | **Lineage** | **Accession Number** | **Reference** |
| --- | --- | --- | --- | --- | --- |
| BD5 | Waters of Baek-du mountain (Paekdu mountain) | North Korea | Subfamily I.1 | KACC13090 (deposited with Korean Agricultural Culture Collection) | (Choi *et al*., 2009) |
| BV26 | For all BioVersys strains- could not find additional info- all just said “from hospital outbreak” | Switzerland (1979) | MLST 1 | [KX819204](http://www-ncbi-nlm-nih-gov.uml.idm.oclc.org/nucleotide/KX819204)(Δ*adeR*)  [KX819214](http://www-ncbi-nlm-nih-gov.uml.idm.oclc.org/nucleotide/KX819214)(Δ*trm*) | (Trebosc *et al*., 2016) |
| BV94 | Not available. | USA (2011) | MLST 2 | [KX819205](http://www-ncbi-nlm-nih-gov.uml.idm.oclc.org/nucleotide/KX819205)(Δ*adeR* )  [KX819215](http://www-ncbi-nlm-nih-gov.uml.idm.oclc.org/nucleotide/KX819215)(Δ*trm*) | (Trebosc *et al*., 2016) |
| BV173 | Not available. | Greece (2012) | MLST 2 | [KX819206](http://www-ncbi-nlm-nih-gov.uml.idm.oclc.org/nucleotide/KX819206)(Δ*adeR*)  [KX819216](http://www-ncbi-nlm-nih-gov.uml.idm.oclc.org/nucleotide/KX819216)(Δ*trm*) | (Trebosc *et al*., 2016) |
| BV175 | Not available. | Turkey (2012) | MLST 2 | [KX819207](http://www-ncbi-nlm-nih-gov.uml.idm.oclc.org/nucleotide/KX819207)(Δ*adeR*)  [KX819217](http://www-ncbi-nlm-nih-gov.uml.idm.oclc.org/nucleotide/KX819217)(Δ*trm*) | (Trebosc *et al*., 2016) |
| BV185 | Not available. | Mexico (2013) | MLST 2 | [KX819208](http://www-ncbi-nlm-nih-gov.uml.idm.oclc.org/nucleotide/KX819208)(Δ*adeR*)  [KX819218](http://www-ncbi-nlm-nih-gov.uml.idm.oclc.org/nucleotide/KX819218)(Δ*trm*) | (Trebosc *et al*., 2016) |
| BV186 | Not available. | USA (2013) | MLST 2 | [KX819209](http://www-ncbi-nlm-nih-gov.uml.idm.oclc.org/nucleotide/KX819209)(Δ*adeR*)  [KX819219](http://www-ncbi-nlm-nih-gov.uml.idm.oclc.org/nucleotide/KX819219)(Δ*trm*) | (Trebosc *et al*., 2016) |
| BV187 | Not available. | USA (2013) | MLST 2 | [KX819210](http://www-ncbi-nlm-nih-gov.uml.idm.oclc.org/nucleotide/KX819210)(Δ*adeR*)  [KX819220](http://www-ncbi-nlm-nih-gov.uml.idm.oclc.org/nucleotide/KX819220) (Δ*trm*) | (Trebosc *et al*., 2016) |
| BV189 | Not available. | Spain (2013) | MLST 2 | [KX819211](http://www-ncbi-nlm-nih-gov.uml.idm.oclc.org/nucleotide/KX819211)(Δ*adeR*)  [KX819221](http://www-ncbi-nlm-nih-gov.uml.idm.oclc.org/nucleotide/KX819221) (Δ*trm*) | (Trebosc *et al*., 2016) |
| **Strain** | **Source** | **Country** | **Lineage** | **Accession Number** | **Reference** |
| BV190 | Not available. | Greece (2012) | MLST 1 | [KX819212](http://www-ncbi-nlm-nih-gov.uml.idm.oclc.org/nucleotide/KX819212)(Δ*adeR*)  [KX819222](http://www-ncbi-nlm-nih-gov.uml.idm.oclc.org/nucleotide/KX819222) (Δ*trm*) | (Trebosc *et al*., 2016) |
| BV191 | Not available. | China (2013) | MLST 2 | [KX819213](http://www-ncbi-nlm-nih-gov.uml.idm.oclc.org/nucleotide/KX819213) (Δ*adeR*)  [KX819223](http://www-ncbi-nlm-nih-gov.uml.idm.oclc.org/nucleotide/KX819223) (Δ*trm*) | (Trebosc *et al*., 2016) |
| AB5075 | Tibia/osteomyelitis, 2008 | USA (US military healthcare system) | Clonal Complex I | MRSN# 959 | (Jacobs *et al*., 2014) |
| AbCAN2 (formerly Ab1225) | Coccygeal | Canada | N/A | CP045428 | (Lopez *et al*., 2020) |
| AYE | Bloodstream infections (in US soldiers)  Urine (Mussi et al., 2011) | France | International Clonal lineage I | CT025832  (EMBL accession number)   NC_010410 (Oh *et al*., 2015) | (Fournier *et al*., 2006) |
| ACICU | Bronchoalveolar lavage  Cerebrospinal Fluid (Mussi et al., 2011) | Italy | International Clonal lineage II (European Clone Group II) | CP000863  NC_010611(Oh *et al*., 2015) | (Iacono *et al*., 2008) |
| Ab_IC I_ | Various clincal specimen in ICU patients | Lithuania | International Clonal lineage I | N/A | (Povilonis *et al*., 2013) |

| **Strain** | **Source** | **Country** | **Lineage** | **Accession Number** | **Reference** |
| --- | --- | --- | --- | --- | --- |
| Ab_IC II_ | Various clinical specimen in ICU patients | Lithuania | International Clonal lineage II | N/A | (Povilonis *et al*., 2013) |
| Ab1 | Blood Culture | Switzerland | International Clonal Lineage I | [LNUY00000000](https://www-ncbi-nlm-nih-gov.uml.idm.oclc.org/nuccore/LNUY00000000) | (Charretier *et al*., 2018) |
| Ab2 | Rectal Swab | Switzerland | International Clonal Lineage II | [LNUZ00000000](https://www-ncbi-nlm-nih-gov.uml.idm.oclc.org/nuccore/LNUZ00000000) | (Charretier *et al*., 2018) |
| Ab3 | Skin Swab | Switzerland | International Clonal Lineage III | [LNUX00000000](https://www-ncbi-nlm-nih-gov.uml.idm.oclc.org/nuccore/LNUX00000000) | (Charretier *et al*., 2018) |
| 1656-2 | Sputum | Korea |  | [CP001921](https://jb-asm-org.uml.idm.oclc.org/lookup/external-ref?link_type=GEN&access_num=CP001921&atom=%2Fjb%2F193%2F22%2F6393.atom).  (Park *et al*., 2011) | (Lee *et al*., 2008) |
| Ab242 | Ascitic Fluid | Argentina | MLST 39 | [DQ309876](https://jb-asm-org.uml.idm.oclc.org/lookup/external-ref?link_type=GEN&access_num=DQ309876&atom=%2Fjb%2F193%2F18%2F4736%2FT1.atom) | (Mussi *et al*., 2011) |
| ABH6 | Unknown | China | N/A | N/A | (Wang *et al*., 2019) |
| XH386 | Pediatric patient | China | ST208 | CP010779 and CP010780 | (Wang *et al*., 2019) |
| BM4454 | Isolated from patient with urinary tract infection | France | N/A | [AF370885](https://aac-asm-org.uml.idm.oclc.org/lookup/external-ref?link_type=GEN&access_num=AF370885&atom=%2Faac%2F45%2F12%2F3375.atom) | (Magnet *et al*., 2001) |
| MDR-ZJ06 | Bloodstream of ICU patient (2006) that suffered from COPD, respiratory failure, and ventilator associated pneumonia | China | European Clone II | [CP001937](https://aac-asm-org.uml.idm.oclc.org/lookup/external-ref?link_type=GEN&access_num=CP001937&atom=%2Faac%2F55%2F10%2F4506.atom) | (Zhou *et al*., 2011) |

| **Strain** | **Source** | **Country** | **Lineage** | **Accession Number** | **Reference** |
| --- | --- | --- | --- | --- | --- |
| HUMC1 | Clinical blood and lung isolate found from an in-patient with ventilator associated pneumonia | USA | MLST2  (Bruhn et al., 2015)  ST206  (Luo et al., 2012) | N/A | (Luo *et al*., 2012) |
| IS-123 | Human Wound | Iraq (2005) | MLST 3 | ALII00000000 | (Chan *et al*., 2011) |

Summary of strains which have been successfully genetically manipulated and are currently being investigated.

**Supplementary Table 2:** Vectors used in genome editing of *A. baumannii*. Use in specific strains has been noted.

| **Vector** | **Selectable Marker(s)** | **Counter-Selection Marker(s)** | **Strain** | **Reference** |
| --- | --- | --- | --- | --- |
| pVT77 | *tpm* | *tdk* , *lacZ* | ATCC17978 BV26, BV94, BV173, BV175, BV185, BV186, BV187, BV189, BV190, BV191 | (Trebosc *et al*., 2016, 2019) |
| pMo130 pMo130-Tel pMo130-Hyg | *aphA kilA-telAB* Hyg^R^ | *sacB* | ATCC17978 BM4454 MDR-ZJ06 | (Hamad *et al*., 2009; Amin *et al*., 2013; Xu *et al*., 2019; De Silva *et al*., 2020) |
| pAT02 | Amp^R^ | N/A | ATCC17978 | (Tucker *et al*., 2014) |
| pCasAb-apr | *aac*(3)-IVa | *sacB* | ABH6 XH386 | (Wang *et al*., 2019) |
| pSGAb-km pSGAb-spe | *aphA*  Sm^R^ | *sacB* | ABH6 XH386 | (Wang *et al*., 2019) |
| pBECAb-apr | *aac*(3)-IVa | *sacB* | ABH6 XH386 | (Wang *et al*., 2019) |
| pColKO | Apr^R^ | *lpxC* | ATCC19606 | (Lee *et al*., 2018) |
| pFLP2^ab^ pFLP2A pFLP2Z | *bla aac*(3)-IVa *ble* | *sacB* | ATCC17978 AB030 AB031 LAC-4 | (Ducas-Mowchun *et al*., 2019) |

*Tpm*; tellurite toxicity, *tdk;* 3’-azido-3’-deoxythymidine (AZT) toxicity, *lacZ*; IPTG inducible, *kilA-telAB*; tellurite toxicity, *aphA;* kanamycin resistance, *sacB;* sucrose toxicity, Hyg^R^; hygromycin resistance, Amp^R^; ampicillin resistance, *aac*(3)-IVa; apramycin resistance, Sm^R^; spectinomycin resistance, Apr^R^; apramycin resistance, *lpxC*; colistin susceptibility, *bla;* β-lactam resistance, *ble;* zeocin resistance

**Supplementary Table 3:** Complementation and cloning vectors used in *A. baumannii.* Use in specific strains has been noted.

| **Vector** | **Selectable Marker(s)** | **Strain** | **Reference** |
| --- | --- | --- | --- |
| pUC18T-miniTn*7*T-Apr pUC18T-miniTn*7*T-Apr-LAC pUC18T-miniTn*7*T-Zeo pUC18T-miniTn*7*T-Zeo-LAC pUC18T-miniTn*7*T-Gm-LAC | *aac*(3)-IVa *lacZ ble* *lacZ aacC1* | ATCC1797 AB030 AB031 LAC-4 AB5075 | (Ducas-Mowchun *et al*., 2019; Pérez-Varela *et al*., 2020, De Silva *et al*., 2020) |
| pYMAb2-Hyg | Hyg^R^ | MDR-ZJ06 | (Xu *et al*., 2019) modified from (Chen *et al*., 2008) |
| pVRL1 pVRL1Z pVRL2 pVRL2Z | *aacC1 tetA ble aacC1 araC* | ATCC19606 ACICU AYE | (Lucidi *et al*., 2018) |
| pABBR-TelR | *kilA-telAB* | Ab Ab2 Ab3 | (Charretier *et al*., 2018) modified from (Tucker *et al*., 2014) |

*aac*(3)-IVa; apramycin resistance, *ble;* zeocin resistance, *lacZ*; IPTG inducible, *aacC1*; gentamicin resistance, *bla;* β-lactamase resistance, Hyg^R;^ hygromycin resistance, *tetA*; tetracycline resistance, *araC*; arabinose inducible, *kilA-telAB;* tellurite toxicity

**Supplementary Table 4**: Fluorescent vectors available for use in *A. baumannii*. Use in specific strains has been noted.

| **Vector** | **Selectable Marker(s)** | **Strain** | **Reference** |
| --- | --- | --- | --- |
| pUC18T-miniTn*7*T-Apr-sfGFP pUC18T-miniTn*7*T-Apr-mCherry pUC18T-miniTn*7*T-Apr-mTurquoise pUC18T-miniTn*7*T-Apr-RubyRed | *aac*(3)-IVa sfGFP mCherry mTurquoise RubyRed | ATCC17978 AB030 LAC-4 | (Ducas-Mowchun *et al*., 2019) |
| pLVP1Z pLVP2Z pLVP3Z | *aacC1*  *ble*  *luxCDABE lacZ* GFP | ATCC19606 ATCC17978 ACICU AYE | (Lucidi *et al*., 2019) |
| pUC18T-mini-Tn*7*T-lux-Ab-dif-apr | *aac*(3)-IVa  *luxCDABE* | UA1Ab | (Jiang *et al*., 2019) |

*aac*(3)-IVa; apramycin resistance, *aacC1*; gentamicin resistance, sfGFP; Green Fluorescent Protein, *ble;* zeocin resistance, *luxCDABE;* operon encoding luminescence, *lacZ;* blue/white selection

Bibliography

Amin, I., Richmond, G. E., Sen, P., Koh, T., Piddock, L. J., and Chua, K. (2013). A Method for Generating Marker-less Gene Deletions in Multidrug-resistant *Acinetobacter baumannii*. *BMC Microbiol.* 13, 158. doi:10.1186/1471-2180-13-158.

Bruhn, K. W., Pantapalangkoor, P., Nielsen, T., Tan, B., Junus, J., Hujer, K. M., et al. (2015). Host Fate is Rapidly Determined by Innate Effector-Microbial Interactions During *Acinetobacter baumannii* Bacteremia. *J. Infect. Dis.* 211, 1296–1305. Available at: https://www.ncbi.nlm.nih.gov/pmc/articles/PMC4447835/ [Accessed July 22, 2020].

Chan, A. P., Sutton, G., DePew, J., Krishnakumar, R., Choi, Y., Huang, X.-Z., et al. (2011). A novel method of consensus pan-chromosome assembly and large-scale comparative analysis reveal the highly flexible pan-genome of *Acinetobacter baumannii*. doi:10.1186/s13059-015-0701-6.

Charretier, Y., Diene, S. M., Baud, D., Chatellier, S., Santiago-Allexant, E., Van Belkum, A., et al. (2018). Colistin Heteroresistance and Involvement of the PmrAB Regulatory System in *Acinetobacter baumannii*. doi:10.1128/AAC.

Chen, T.-L., Chen-Chih Wu, R., Shaio, M.-F., Fung, C.-P., and Cho, W.-L. (2008). Acquisition of a Plasmid-Borne bla OXA-58 Gene with an Upstream IS1008 Insertion Conferring a High Level of Carbapenem Resistance to *Acinetobacter baumannii*. *Antimicrob. Agents Chemother.* 52, 2573–2580. doi:10.1128/AAC.00393-08.

De Silva, P. M., Patidar, R., Graham, C. I., Karen, A., Brassinga, C., and Kumar, A. (2020). A response regulator protein with antar domain, AvnR, in *Acinetobacter baumannii* ATCC 17978 impacts its virulence and amino acid metabolism. *Microbiology* 166, 554–566. doi:10.1099/mic.0.000913.

Ducas-Mowchun, K., Malaka, P., Silva, D., Crisostomo, L., Fernando, D. M., Chao, T.-C., et al. (2019). Next Generation of Tn*7*-Based Single-Copy Insertion Elements for Use in Multi- and Pan-Drug-Resistant Strains of *Acinetobacter baumannii*. *Appl. Environ. Microbiol.* 85, e00066-19. doi:doi: 10.1128/AEM.00066-19.

Fernando, D., Zhanel, G., and Kumar, A. (2013). Antibiotic Resistance and Expression of Resistance-Nodulation-Division Pump- and Outer Membrane Porin-Encoding Genes in *Acinetobacter* species Isolated from Canadian Hospitals. *Can J Infect Dis Med Microbiol* 24, 17–21. Available at: https://www.ncbi.nlm.nih.gov/pmc/articles/PMC3630023/pdf/jidmm24017.pdf [Accessed April 25, 2018].

Fournier, P. E., Vallenet, D., Barbe, V., Audic, S., Ogata, H., Poirel, L., et al. (2006). Comparative genomics of multidrug resistance in *Acinetobacter baumannii*. *PLoS Genet.* 2, 62–72. doi:10.1371/journal.pgen.0020007.

Hamad, M. A., Zajdowicz, S. L., Holmes, R. K., and Voskuil, M. I. (2009). An Allelic Exchange System for Compliant Genetic Manipulation of the Select Agents *Burkholderia pseudomallei* and Burkholderia mallei. *Gene* 430, 123–131. doi:10.1016/j.gene.2008.10.011.

Iacono, M., Villa, L., Fortini, D., Bordoni, R., Imperi, F., Bonnal, R. J. P., et al. (2008). Whole-genome pyrosequencing of an epidemic multidrug-resistant *Acinetobacter baumannii* strain belonging to the European clone II group. *Antimicrob. Agents Chemother.* 52, 2616–2625. doi:10.1128/AAC.01643-07.

Jiang, H., Gao, Y., Zeng, S., Wang, S., Cao, Z., Tan, Y., et al. (2019). One-Step Engineering of a Stable, Selectable Marker-Free Autoluminescent *Acinetobacter baumannii* for Rapid Continuous Assessment of Drug Activity S. *J. Microbiol. Biotechnol.* 29, 1488–1493. doi:10.4014/jmb.1905.05006.

Lee, W., Do, T., Zhang, G., Kahne, D., Meredith, T. C., and Walker, S. (2018). Antibiotic Combinations That Enable One-Step, Targeted Mutagenesis of Chromosomal Genes. *ACS Infect. Dis.* 4, 1007–1018. doi:10.1021/acsinfecdis.8b00017.

Lopez, J., Ly, P. M., and Feldman, M. F. (2020). The Tip of the VgrG Spike Is Essential to Functional Type VI Secretion System Assembly in *Acinetobacter baumannii*. *MBio* 11. doi:10.1128/mBio.02761-19.

Lucidi, M., Runci, F., Rampioni, G., Frangipani, E., Leoni, L., and Visca, P. (2018). New Shuttle Vectors for Gene Cloning and Expression in Multidrug-Resistant *Acinetobacter* Species. doi:10.1128/AAC.02480-17.

Lucidi, M., Visaggio, D., Prencipe, E., Imperi, F., Rampioni, G., Cincotti, G., et al. (2019). New shuttle vectors for real-time gene expression analysis in multidrug-resistant *Acinetobacter species*: In vitro and in vivo responses to environmental stressors. *Appl. Environ. Microbiol.* 85, e01334-19. doi:10.1128/AEM.01334-19.

Luo, G., Lin, L., Ibrahim, A. S., Baquir, B., Pantapalangkoor, P., Bonomo, R. A., et al. (2012). Active and Passive Immunization Protects against Lethal, Extreme Drug Resistant-*Acinetobacter baumannii* Infection. *PLoS One* 7, e29446. doi:10.1371/journal.pone.0029446.

Mussi, M. A., Limansky, A. S., Relling, V., Ravasi, P., Arakaki, A., Actis, L. A., et al. (2011). Horizontal gene transfer and assortative recombination within the *Acinetobacter baumannii* clinical population provide genetic diversity at the single carO gene, encoding a major outer membrane protein channel. *J. Bacteriol.* 193, 4736–4748. doi:10.1128/JB.01533-10.

Pérez-Varela, M., Tierney, A. R. P., Kim, J.-S., Vázquez-Torres, A., and Rather, P. (2020). Characterization of RelA in *Acinetobacter baumannii*. *J. Bacteriol.* 202, e00045-20. doi:10.1128/JB.00045-20.

Povilonis, J., Seputiene, V., Krasauskas, R., Juškaite, R., Miškinyte, M., Sužiede, S., et al. (2013). Spread of carbapenem-resistant *Acinetobacter baumannii* carrying a plasmid with two genes encoding OXA-72 carbapenemase in Lithuanian hospitals. *J. Antimicrob. Chemother.* 68, 1000–1006. doi:10.1093/jac/dks499.

Sato, Y., Unno, Y., Ubagai, T., and Ono, Y. (2018). Sub-minimum inhibitory concentrations of colistin and polymyxin B promote *Acinetobacter baumannii* biofilm formation. *PLoS One* 13, e0194556. doi:10.1371/journal.pone.0194556.

Singh, M., De Silva, P. M., Al-Saadi, Y., Switala, J., Loewen, P. C., Hausner, G., et al. (2020). Antibiotics Characterization of Extremely Drug-Resistant and Hypervirulent *Acinetobacter baumannii* AB030. *Antibiot.* 9. doi:10.3390/antibiotics9060328.

Trebosc, V., Gartenmann, S., Royet, K., Manfredi, P., Tötzl, M., Schellhorn, B., et al. (2016). A Novel Genome-Editing Platform for Drug-Resistant *Acinetobacter baumannii* Reveals an AdeR-Unrelated Tigecycline Resistance Mechanism. *Antimicrob. Agents Chemother.* 60, 7263–7271. doi:10.1128/AAC.01275-16.

Trebosc, V., Gartenmann, S., Tötzl, M., Lucchini, V., Schellhorn, B., Pieren, M., et al. (2019). Dissecting Colistin Resistance Mechanisms in Extensively Drug-Resistant *Acinetobacter baumannii* Clinical Isolates. *MBio* 10, e01083-19. doi:10.1128/mBio.

Tucker, A. T., Nowicki, E. M., Boll, J. M., Knauf, G. A., Burdis, N. C., Trent, M. S., et al. (2014). Defining gene-phenotype relationships in *Acinetobacter baumannii* through one-step chromosomal gene inactivation. *MBio* 5, 1313–1327. doi:10.1128/mBio.01313-14.

Wang, Y., Wang, Z., Chen, Y., Hua, X., Yu, Y., and Correspondence, J. (2019). A Highly Efficient CRISPR-Cas9-Based Genome Engineering Platform in *Acinetobacter baumannii* to Understand the H 2 O 2-Sensing Mechanism of OxyR. *Cell Chem. Biol.* 26, 1732–1742. doi:10.1016/j.chembiol.2019.09.003.

Xu, C., Bilya, S. R., and Xu, W. (2019a). adeABC efflux gene in *Acinetobacter baumannii*. *New Microbes New Infect.* 30. doi:10.1016/j.nmni.2019.100549.

Xu, Q., Chen, T., Yan, B., Zhang, L., Pi, B., Yang, Y., et al. (2019b). Dual Role of gnaA in Antibiotic Resistance and Virulence in *Acinetobacter baumannii*. *Antimicrob. Agents Chemother.* 63, e00694-19. doi:doi: 10.1128/AAC.00694-19.

Zhu, Y., Lu, J., Zhao, J., Zhang, X., Yu, H. H., Velkov, T., et al. (2020). Complete genome sequence and genome-scale metabolic modelling of *Acinetobacter baumannii* type strain ATCC 19606. *Int. J. Med. Microbiol.* 310, 151412. doi:10.1016/j.ijmm.2020.151412.
